# Supplementary material for: Awareness-driven Behavior Changes Can Shift the Shape of Epidemics Away from Peaks and Towards Plateaus, Shoulders, and Oscillations
Source: medRxiv. 2020 Oct 16:2020.05.03.20089524. Preprint. [Version 3] doi: 10.1101/2020.05.03.20089524 (PMC7273247; doi:10.1101/2020.05.03.20089524)
Supplement: 1 [file NIHPP2020.05.03.20089524-supplement-1.pdf]

medRxiv preprint doi: https://doi.org/10.1101/2020.05.03.20089524; this version posted October 16, 2020. The copyright holder for this preprint (which was not certified by peer review) is the author/funder, who has granted medRxiv a license to display the preprint in perpetuity.
It is made available under a CC-BY 4.0 International license .

Appendix A: Appendix - Supplementary Information

medRxiv preprint doi: https://doi.org/10.1101/2020.05.03.20089524; this version posted October 16, 2020. The copyright holder for this preprint (which was not certified by peer review) is the author/funder, who has granted medRxiv a license to display the preprint in perpetuity.
It is made available under a CC-BY 4.0 International license .

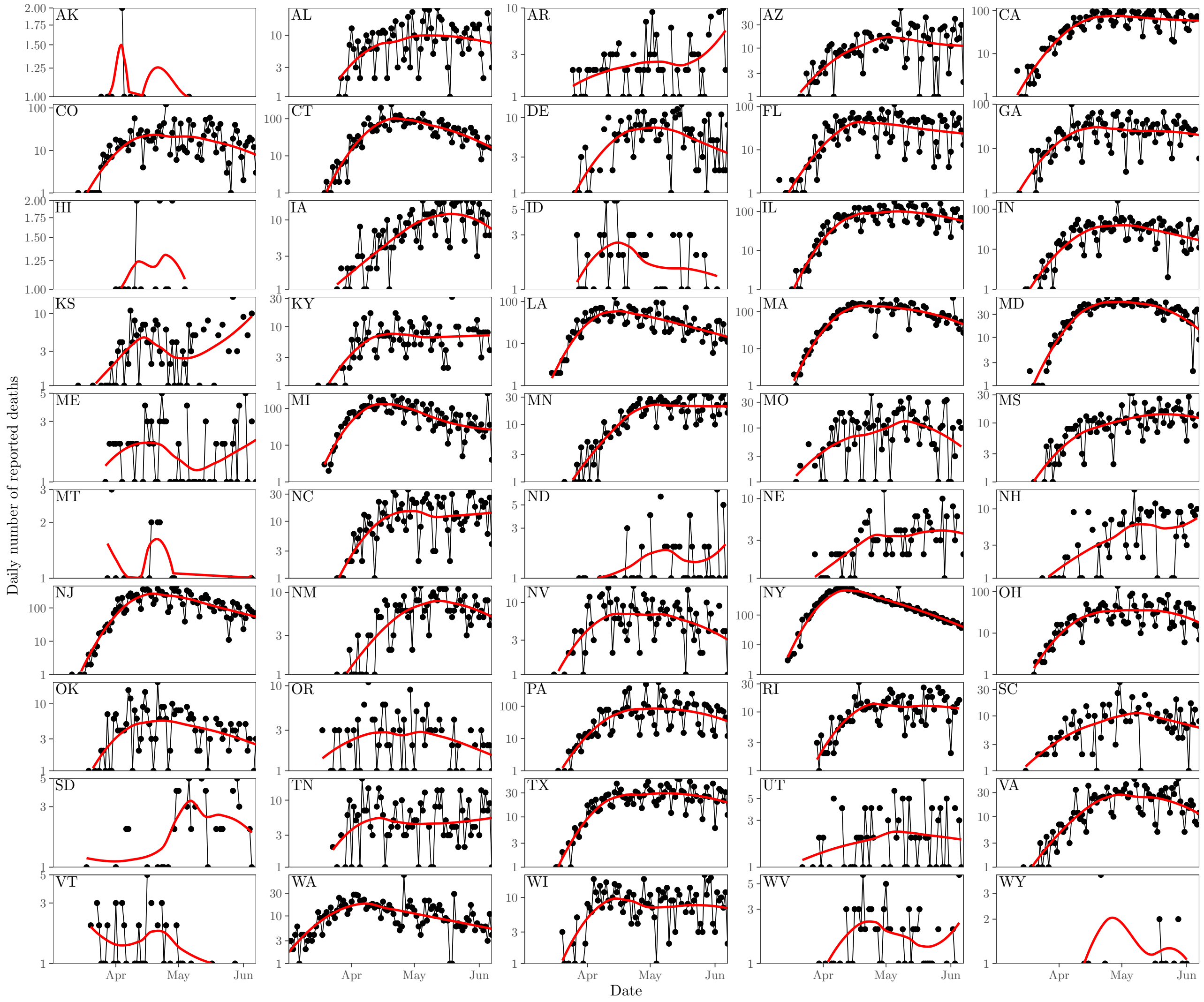

FIG. S1: Daily number of reported deaths for COVID-19 (black points and lines) and the corresponding locally estimated scatterplot smoothing (LOESS) curves (red lines) in 50 states.

medRxiv preprint doi: https://doi.org/10.1101/2020.05.03.20089524; this version posted October 16, 2020. The copyright holder for this preprint (which was not certified by peer review) is the author/funder, who has granted medRxiv a license to display the preprint in perpetuity.
It is made available under a CC-BY 4.0 International license .

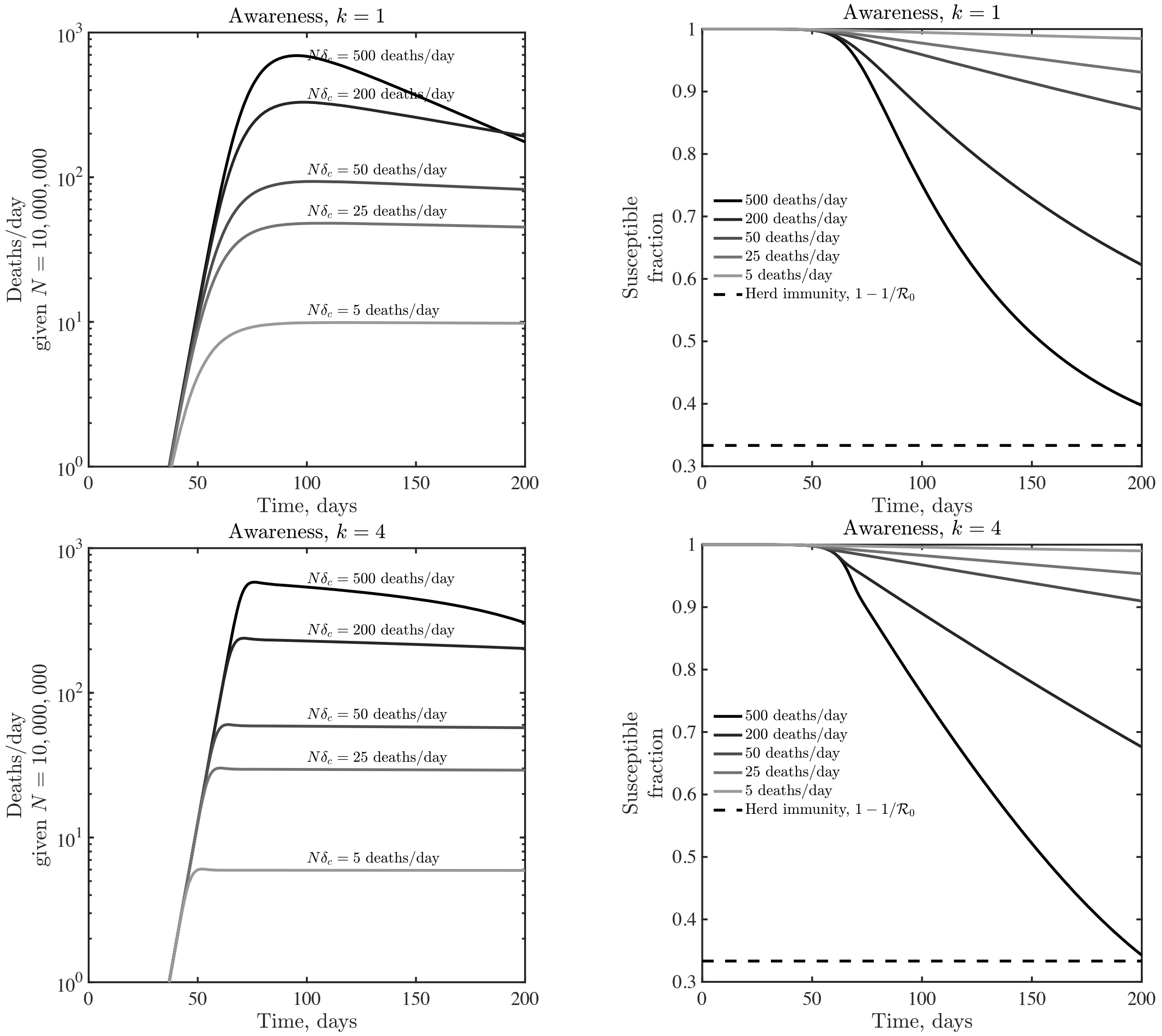

FIG. S2: Dynamics given variation in the critical fatality awareness level, δ_c for awareness $k = 1$ (top) and $k = 4$ (bottom). Panels show deaths/day (top) and the susceptible fraction as a function of time (bottom), the latter compared to a herd immunity level when only a fraction $1/\mathcal{R}_0$ remain susecptible. These simulations share the epidemiological parameters $\beta = 0.5$ /day, $\mu = 1/2$ /day, $\gamma = 1/6$ /day, and $f_D = 0.01$.

medRxiv preprint doi: https://doi.org/10.1101/2020.05.03.20089524; this version posted October 16, 2020. The copyright holder for this preprint
(which was not certified by peer review) is the author/funder, who has granted medRxiv a license to display the preprint in perpetuity.
It is made available under a CC-BY 4.0 International license .

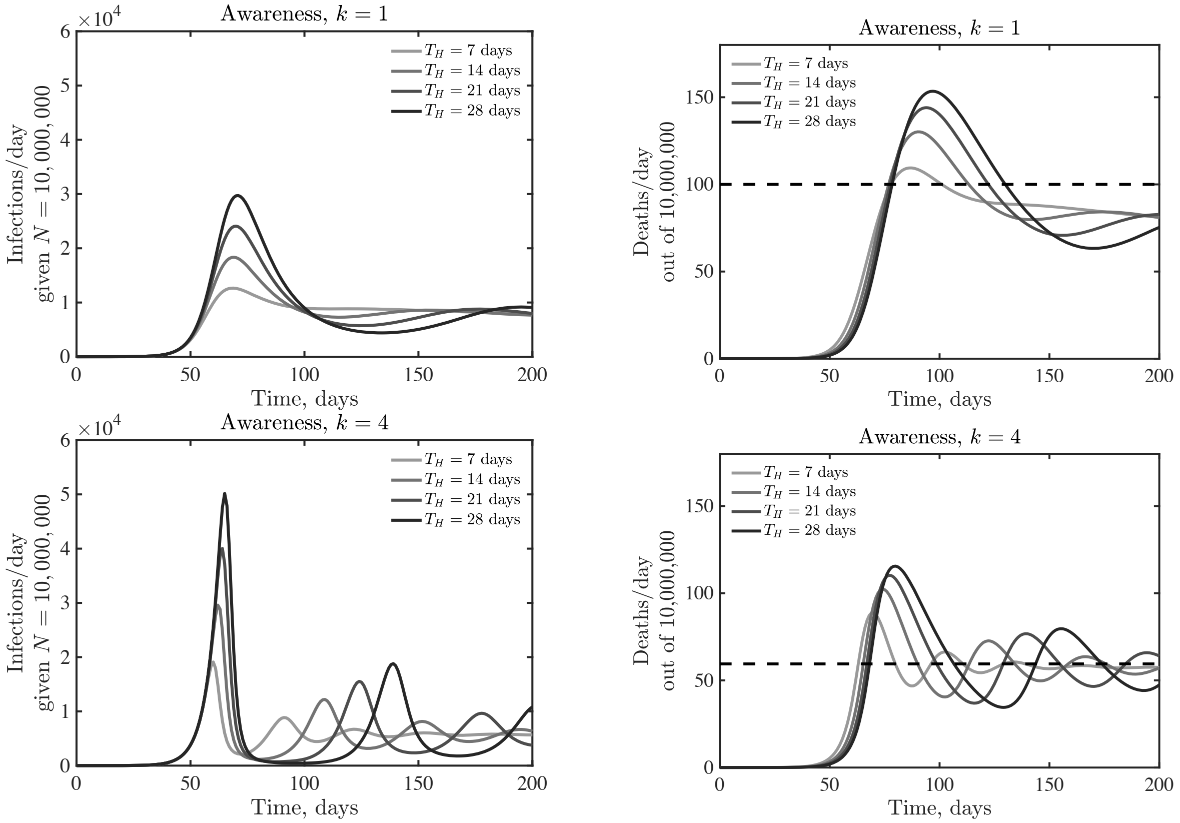

FIG. S3: Emergence of oscillatory dynamics in a death-driven awareness model of social distancing given lags between infection and fatality. Awareness is $k = 1$ (top) and $k = 4$ (bottom), all other parameters as in Figure 3. The dashed lines for fatalities expected quasi-stationary value $\delta^{(q)}$.

medRxiv preprint doi: https://doi.org/10.1101/2020.05.03.20089524; this version posted October 16, 2020. The copyright holder for this preprint (which was not certified by peer review) is the author/funder, who has granted medRxiv a license to display the preprint in perpetuity.
It is made available under a CC-BY 4.0 International license .

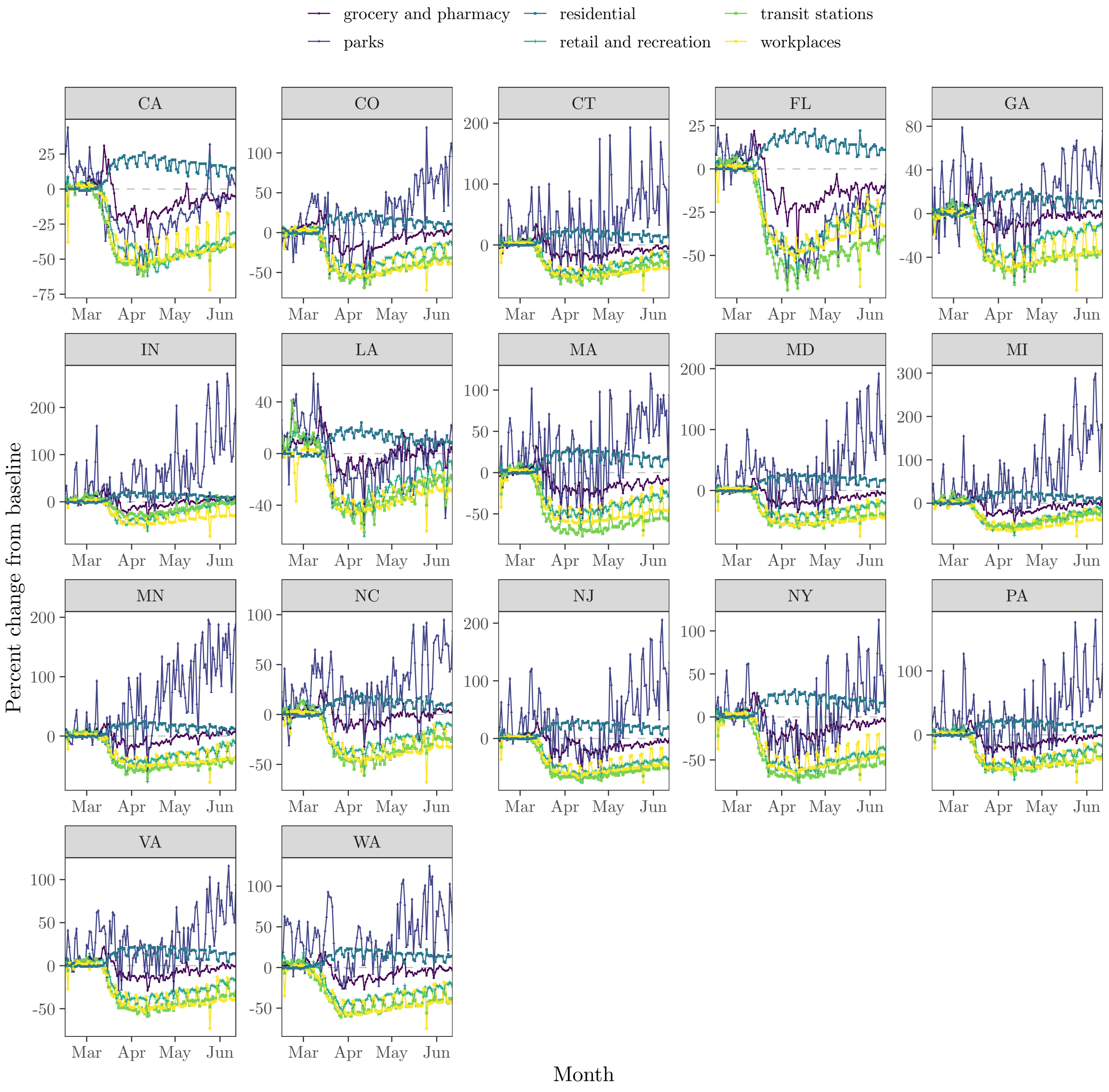

FIG. S4: Percent mobility change from baseline across six categories in 17 states.
